# Amaranth Plants with Various Color Phenotypes Recruit Different Soil Microorganisms in the Rhizosphere

**DOI:** 10.3390/plants13162200

**Published:** 2024-08-08

**Authors:** Xin-Ru Lin, Da Yang, Yu-Fei Wei, Dian-Cao Ding, Hui-Ping Ou, Shang-Dong Yang

**Affiliations:** 1 Guangxi Key Laboratory of Agro-Environment and Agro-Products Safety, National Demonstration Center for Experimental Plant Science Education Guangxi Agricultural College, Guangxi University, Nanning 530004, China; lxr946785@163.com (X.-R.L.);; 2 Agricultural Resources and Environmental Research Institute, Guangxi Academy of Agricultural Sciences/Guangxi Key Laboratory of Arable Land Conservation, Nanning 530004, China

**Keywords:** Amaranthus (*Amaranthus tricolor* L.), color, rhizospheric bacteria, rhizospheric fungi

## Abstract

To explore and utilize the abundant soil microorganisms and their beneficial functions, high-throughput sequencing technology was used to analyze soil microbial compositions in the rhizosphere of red and green amaranth varieties. The results showed that significant differences in soil microbial composition could be found in the rhizosphere of amaranth plants with different color phenotypes. Firstly, soil bacterial compositions in the rhizosphere were significantly different between red and green amaranths. Among them, *Streptomyces, Pseudonocardia*, *Pseudolabrys*, *Acidibacter, norank_ f_ Micropepsaceae*, *Bradyrhizobium*, and *Nocardioides* were the unique dominant soil bacterial genera in the rhizosphere of red amaranth. In contrast, *Conexibacter*, *norank_f_norank_o_norank_c_TK10*, and *norank_f_ norank_o_ norank_ c_AD3* were the special dominant soil bacterial genera in the rhizosphere of green amaranth. Additionally, even though the soil fungal compositions in the rhizosphere were not significantly different between red and green amaranths, the abundance of the dominant soil fungal genera in the rhizosphere showed significant differences between red and green amaranths. For example, *unclassified_k__Fungi*, *Fusarium*, *Cladophialophora*, *unclassified_c__Sordariomycetes* and *unclassified_p__Chytridiomycota* significantly enriched as the dominant soil fungal genera in the rhizosphere of the red amaranth. In contrast, *Aspergillues* only significantly enriched as the dominant soil fungal genus in the rhizosphere of green amaranth. All of the above results indicated that amaranth with various color phenotypes exactly recruited different microorganisms in rhizosphere, and the enrichments of soil microorganisms in the rhizosphere could be speculated in contributing to amaranth color formations.

## 1. Introduction

Amaranth (*Amaranthus tricolor* L.) is an annual herbaceous plant of the Amaranthaceae family that originated in the Americas and Asia, and now grows widely in Asia, the Americas, and Europe [1,2]. It is rich in carotenoids, vitamins, and minerals as well as many other substances beneficial to human health [3,4]. Amaranth plants exhibit a variety of colors, which is one of their most important commercial qualities. Different based on color classification, amaranth can be classified as red or green [5,6]. Different colored amaranth species have different nutritional values. Among them, red amaranth plants contain more nutrients than green amaranth plants [7]. Therefore, it is of great significance to explore the formation mechanism of amaranth color.

Generally speaking, the color of plants mainly depends on the pigment species and content in the plant body. These pigments mainly include chlorophyll, carotenoids, and flavonoids (such as anthocyanins) [8,9,10]. Among them, the different colors of red amaranth and green amaranth are mainly due to the different betalain content in plants. In comparison with green amaranth, the content of betalain in the leaves of beet and red amaranth was significantly higher. Therefore, it can make amaranth appear red [11,12,13]. Betaine is a water-soluble pigment, mainly divided into betacyanin and betaxanthin [14,15], which is widely found in the roots, stems, and leaves of plants [16,17,18]. However, the biosynthesis of betalain as a secondary metabolite is affected by many factors, such as light, temperature, humidity, and hormone levels, especially phytohormones [19,20,21]. For example, salicylic and jasmonic acids significantly promote betaine synthesis [22]. In contrast, gibberellin inhibits amaranth pigment biosynthesis [23]. Moreover, betaine can also be produced by microorganisms, such as the fungal lineage and some diazotrophic gluconic isolate bacteria [24].

Soil microorganisms in the rhizosphere are the most active component of the soil-plant interaction system and are considered the second most common type of genome in plants [25,26]. A large number of studies have shown that rhizospheric soil microorganisms participate not only in material circulation but also in energy metabolism, which plays an important role in maintaining soil fertility, health, and sustainable agricultural production [27,28,29]. For example, Abinandan et al. [30] found that soil microorganisms play an important role in maintaining nutrient balances, carbon sinks, and soil health. Meanwhile, Priya et al. [31] also found that the state of soil health and its nutrient pools are dependent on soil microbial community structure and function. In addition, rhizospheric microorganisms can also directly or indirectly affect the growth and development of crops [32,33]. The direct mechanism involved microbes in the rhizosphere producing plant hormones or enzymes, which regulate the growth and development of plants [34]. For example, auxin [35], gibberellin (GA) [36], cytokinin [37], and ethylene [38] can be produced by microbes in the rhizosphere to improve their adaptability. An indirect mechanism involves the participation of soil microbes in defensive metabolic processes, plant pathogen inhibition, and environmental stresses in the rhizosphere [39].

Like all higher organisms, plants have evolved in the context of the microbial world. For instance, microorganisms co-evolved with their hosts and co-existing microbial consortia to expand the host’s genetic pool, known as “extended genotypes” [40]. The hosts can integrate the expanded microbial genomes into their phenotype, resulting in profound changes in phenotypic characteristics, the “extended phenotype” [41]. However, whether different genotypes of amaranth recruit different rhizosphere microorganisms by controlling the secretion of certain substances (enzymes, hormones, etc.) to promote the formation of their own colors has not been explored before. Therefore, we hypothesized that amaranth species with different color phenotypes would recruit different microbes in synthesizing their color. To test this hypothesis, the soil microbial compositions in the rhizospheres of red and green amaranths were analyzed using a high-throughput sequencing technique.

## 2. Methods

### 2.1. Field Site Description and Experimental Design

Two different colored amaranths were used in this study. First, red amaranth varieties (R), which included “a little red” (Qingxian Xingyun Vegetable Breeding Co., Ltd., Cangzhou, China) and “bonus Garden Leaf” (Qiangkun Vegetable Seed Co., Ltd., Qinzhou, China), were used. Green amaranth varieties (G), including “green amaranth” (Jiuquan Changfeng Agricultural Development Co., Ltd., Jiuquan, China) and “Qingyuan Leaf” (Guangzhou Hongye Seed Technology Co., Ltd., Guangzhou, China), were also used for comparative study.

The experiment was carried out in pots with a diameter of 32 cm and a height of 20 cm from December 2021 to June 2022 at the vegetable base of the College of Agriculture, Guangxi University (108°17′15″ E, 22°51′02″ N); 30 pots were planted for each amaranth variety. The soil type was red loam, and all the amaranth varieties were identically treated. The study area is a typical subtropical monsoon climate, with abundant precipitation, long summer, and short winter. The average annual air temperature and rainfall were 21.8 °C and 1286 mm, respectively. The physicochemical properties of the soil were as follows: pH 5.71; organic matter content, 8.42 g·kg^−1^; and total nitrogen, phosphorus, and potassium, 0.51 g·kg^−1^, 0.67 g·kg^−1^, and 7.21 g·kg^−1^, respectively. Moreover, the available phosphorus, potassium, and alkaline nitrogen contents were 0.59 mg·kg^−1^, 51.01 mg·kg^−1^, and 13.17 mg·kg^−1^, respectively.

### 2.2. Soil Sampling

After the amaranth had reached maturity, three uniformly growing amaranth plants were selected for each variety, and rhizospheric soil samples of the amaranth were collected using the root-shaking method [42]. Specifically, the shovel was disinfected with alcohol and used to loosen the soil around the amaranth plant; then, the whole amaranth plant was poured out and shaken, and the soil adhering to the root of the plant was collected as rhizospheric soil samples. The plants were put into sterile bags, brought back to the laboratory, screened through 10 mesh, and subjected to microbial community analysis. In addition, soil without any plants was also collected and used as a bulk (CK) soil.

### 2.3. Soil Microbial DNA Extraction and Illumina Sequencing

The soil microbial community structures in the rhizosphere of amaranths of various color phenotypes were sequenced by Shanghai Meiji Biomedical Technology Co.(Shanghai, China) Total DNA was extracted according to the instructions of the E.Z.N.A.A. Soil DNA kit (Omega, Norcross, GA, USA), DNA concentration and purity were detected using a NanoDrop2000 spectrophotometer (Thermo, Wilmington, DE, USA), and extracted genomic DNA was detected using 1% agarose gel electrophoresis. The extracted interrhizosphere soil microbial DNA was used as a template. The primers 338F (5′-ACTCCTACGGGAGGCAGCAG-3′) and 806R (5′-GGACTACHVGGGTWTCTAAT-3′) were selected for PCR amplification of the 16S rRNA V5-V7 region of interrhizosphere soil bacteria, and the primer ITS1F (5′-CTTGGTCATTTAGAGGA) was selected for PCR amplification of the 16S rRNA V5-V7 region of interrhizosphere soil bacteria. CTTGGTCATTTAGAGGAAGTAA-3′) and ITS2F (5′-GCTGCGTTCTTCATCATCGATFC-3′) were selected for PCR amplification of the 18S rRNA ITS region of the interrhizosphere soil fungus, and the PCR instrument used was an ABI GeneAmp^®^ 9700 model. The PCR products were recovered by 2% agarose gel electrophoresis, purified using an Axy PrepDNA Gel Recovery Kit (Axygen, San Francisco, CA, USA), and eluted with Tris_HCl. The PCR products were detected and quantified using a QuantiFluor™-ST Blue Fluorescence Quantification System (Promega, Madison, WI, USA). The purified amplified fragments were used to construct libraries according to the standard protocols of the Illumina MiSeq platform.

### 2.4. Data Analysis

The experimental data were statistically analyzed using Excel 2019 and SPSS 221.0, and Duncan’s multiple range test was used to compare the means. The alpha diversity of the bacterial and fungal communities was calculated using Mothur (version v.1.30.2; https://mothur.org/wiki/calculators/, accessed on 9 April 2022). Non-metric multidimensional scaling (NMDS) and partial least squares discriminant analysis (PLS-DA) were used for statistical analysis and mapping, respectively, using R language (version 3.3.1) tools. For microbial community composition and Venn diagram analysis, Operational taxonomic units (OTU) tables with 97% similarity were selected and used for statistical and mapping purposes via the R language (version 3.3.1) tool. The linear discriminant analysis effect size (LEfSe) method was used to identify the soil microbial community structures in the rhizosphere. Network correlation analysis of rhizospheric soils between red and green amaranths was carried out using correlation coefficient Spearman [43]. Second, PICRUSt, the Kyoto Encyclopedia of Genes and Genomes (KEGG) dataset, was used to estimate the functional composition of the bacterial community. Additionally, the functional prediction of fungal communities was also evaluated using the Fungi Functional Association (FUNGuild) tool. All the online data analyses were conducted using the free online platform Majorbio Cloud Platform (www.majorbio.com, accessed on 8 August 2024) through Majorbio Biopharm Technology Co., Ltd., (Shanghai, China). The data were visualized by ImageGP (https://onlinelibrary.wiley.com/doi/10.1002/imt2.5, accessed on 9 April 2022).

## 3. Results

### 3.1. Analysis of Soil Bacterial and Fungi Alpha Diversity in the Rhizosphere of Red and Green Amaranth Plants

As shown in Table 1, the soil bacterial diversity (Shannon and Simpson) and richness (Ace and Chao1) in the rhizospheres of red and green amaranths were all not significantly different between each other. Also, there were no significant differences with bulk soil. Additionally, soil fungi diversity (Shannon and Simpson) and richness (Ace and Chao1) in the rhizospheres of red and green amaranths were also not significantly different between each other, and there were no significant differences between bulk soil either.

### 3.2. Analysis of Soil Bacterial and Fungi Beta Diversity in the Rhizospheres of Red and Green Amaranth Plants

**Figure 1:**
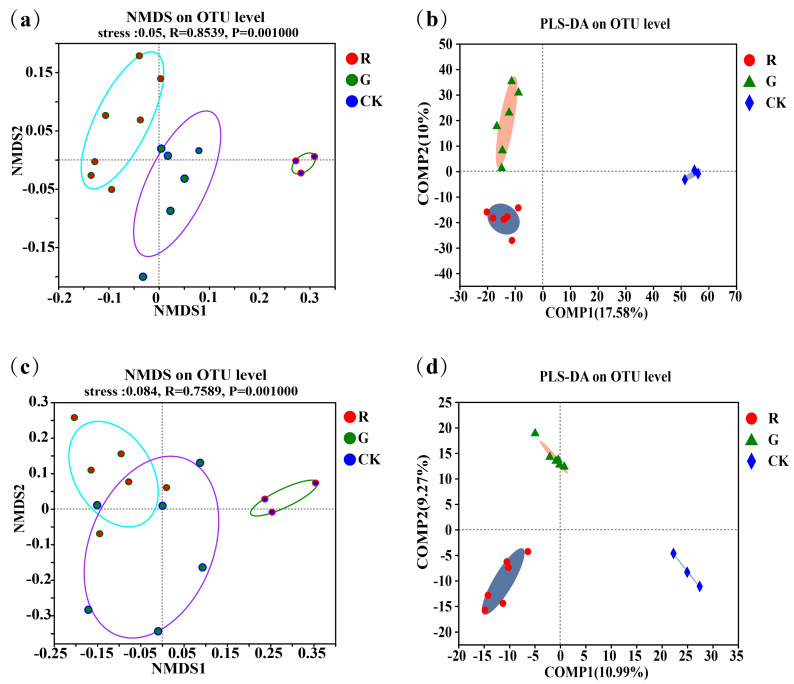
Comparison of soil bacterial communities in the rhizospheres of red and green amaranth plants; (**a**) the non-metric multidimensional scaling (NMDS) of soil bacteria in the rhizospheres of red and green amaranth plants at the operational taxonomic unit (OTU) level; (**b**) the partial least squares discriminant analysis (PLS-DA) of soil bacteria in the rhizospheres of red and green amaranth plants at the operational taxonomic unit (OTU) level; (**c**) the non-metric multidimensional scaling (NMDS) of soil fungi in the rhizospheres of red and green amaranth plants at the operational taxonomic unit (OTU) level; (**d**) the partial least squares discriminant analysis (PLS-DA) of soil fungi in the rhizospheres of red and green amaranth plants at the operational taxonomic unit (OTU) level.

Non-metric multidimensional scaling (NMDS) analyses of the soil microbial community structures in the rhizospheres of red and green amaranths were carried out at the operational taxonomic unit (OTU) level (Figure 1a,c). The results showed that not only soil bacterial (R = 0.8539, *p* = 0.001) but also soil fungal (R = 0.7589, *p* = 0.001) community structures in the rhizospheres were all significantly different in the rhizospheres between red and green amaranths. In addition, we also performed partial least squares discriminant analysis (PLS-DA) at the operational taxonomic unit (OTU) level (Figure 1b). The results also showed that soil bacteria and fungi clustered separately in the rhizosphere between red and green amaranths, respectively. All of the above results indicated that different soil microbes were exactly recruited in the rhizospheres of red and green amaranths even though they were grown in the same field under identical management.

### 3.3. Community Composition Analysis of Soil Bacteria and Fungi in the Rhizospheres of Red and Green Amaranth Plants

**Figure 2:**
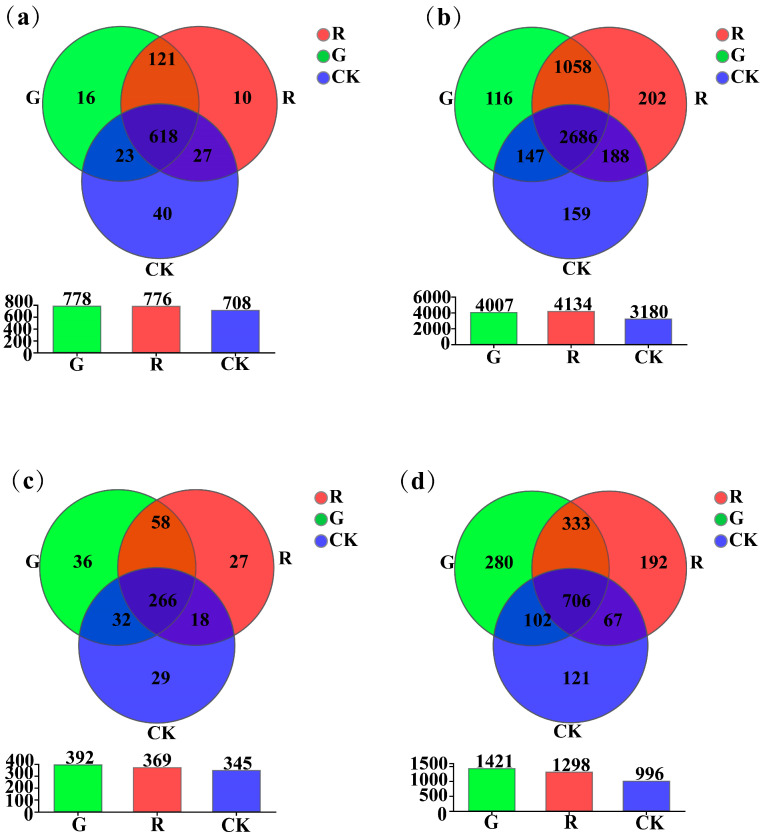
Venn plot analysis of soil bacteria (**a**,**b**) and fungi (**c**,**d**) in the rhizosphere of red and green amaranths at the genus and operational taxonomic unit (OTU) levels.

As shown in Figure 2, at the genus level, the results of Venn diagram analyses showed that the numbers of unique soil dominant bacterial genera in the rhizospheres of red and green amaranths, and the bulk soil were 10, 16, and 40, respectively (Figure 2a). However, at the operational taxonomic unit (OTU) level, they were 202, 116, and 159, respectively (Figure 2b). For soil fungi, at the genus level, the results of Venn diagram analyses also showed that the numbers of unique soil dominant fungal genera in the rhizosphere of red amaranth, green amaranth, and bulk soil (CK) were 27, 36, and 27, respectively (Figure 2c). Moreover, at the operational taxonomic unit (OTU) level, they were 192, 280, and 121, respectively (Figure 2d).

**Figure 3:**
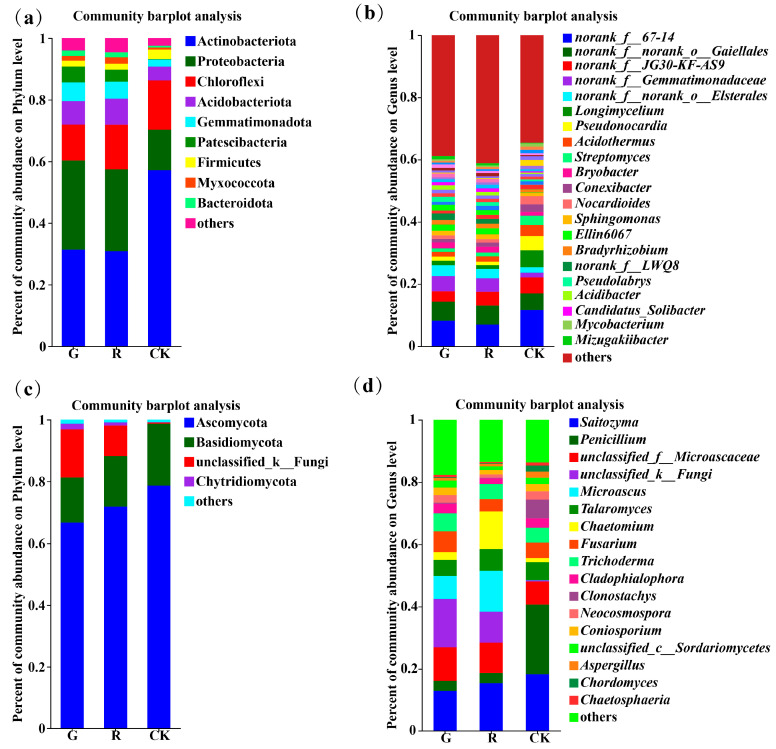
Composition of soil bacterial communities in the rhizosphere of red and green amaranth plants at the phylum (**a**) and genus (**b**) levels. Composition of soil fungi communities in the rhizosphere of red and green amaranth plants at the phylum (**c**) and genus (**d**) levels.

At the phylum level, there were 9, 9, and 6 dominant soil bacterial phyla (with an abundance greater than 1%) in the rhizosphere of red and green amaranths and bulk soil, respectively (Figure 3a). Among them, the dominant bacterial phyla in the rhizosphere of red amaranth were Actinobacteriota (31.37%), Proteobacteria (28.91%), Chloroflexi (11.65%), Acidobacteriota (7.62%), Gemmatimonadota (6.09%), Patescibacteria (5.17%), Firmicutes (1.92%), Myxococcota (1.59%), and Bacteroidota (1.79%). In contrast, the dominant soil bacterial phyla in the rhizosphere of green amaranth were Actinobacteriota (30.87%), Proteobacteria (26.54%), Chloroflexi (14.5%), Acidobacteriota (8.46%), Gemmatimonadota (5.52%), Patescibacteria (3.92%), Myxococcota (2. 05%), Firmicutes (1.85%), and Bacteroidota (1.64%). Moreover, the dominant bacterial phyla of the bulk soil were Actinobacteria (57.14%), Proteobacteria (13.19%), Chloroflexi (11.65%), Acidobacteriota (4.50%), Gemmatimonadota (2.27%), and Firmicutes (3.07%).

At the genus level, the numbers of dominant soil bacterial genera (those with an abundance greater than 1%) in the rhizosphere of red and green amaranths and bulk soil were 18, 17, and 18, respectively (Figure 3b).

First, *norank_f_67-14* (8.25%), *norank_f_norank_o_Gaiellales* (6.06%), norank_f_ *Gemmatimonadaceae* (4.93%), *norank_f_norank_o_Elsterales* (3.47%), *norank_f_JG30-KF-AS9* (3.32%), *Streptomyces* (2.91%), *Ellin6067* (2.00%), *norank_LWQ8* (2.11%), *Bryobucter* (1.94%), *unclassified_o_Saccharimonadales* (1.88%), *Pseudolabrps* (1.59%), *Sphingomons* (1.51%), *Acidothermus* (1.50%), *Longimycelium* (1.47%), *Acidibacter* (1.46%), *Bradyrhizobium* (1.45%), *Pseudonocardia* (1.33%), *norank_f_Micropepsaceae* (1.28%), and *Nocardioides* (1.28%) were the dominant soil bacterial genera in the rhizosphere of red amaranth.

In contrast, *norank_f_67-14* (6.94%), *norank_f_ norank_o_Gaiellales* (6.14%), *norank_f_JG30-KF-AS9* (4.41%), *norank_f_ Gemmatimonadaceae* (4.35%), *norank_f_ norank_o_Elsterales* (3.01%), *Ellin6067* (1.92%), *Bryobacter* (1.89%), *Acidothermus* (1.75%), *Sphingomonas* (1.62%), norank_f_LWQ8 (1.53%), *unclassified_o_Saccharimonadales* (1.50%), norank_f_ norank_o_ norank_ c_ AD3 (1.35%), *norank_f_norank_o _norank _c_ TK10* (1.34%), *Comexibacter* (1.30%), *Longimyelium* (1.22%), and *Pseudolabrys* (1.21%) were the dominant soil bacterial genera in the rhizosphere of green amaranth.

Moreover, *norank_f_67-14* (11.67%), *Longimycelium* (5.44%), *norank_f_ norank_o_Gaiellales* (5.33%), *norank_f_JG30-KF-AS9* (5.14%), *Pseudonocardia* (4.55%), *Acidothermus* (3.60%), *Nocardioides* (2.59%), *Conexibacter* (2.46%), *Actinoallomurus* (1.82%), *norank_f_norank_o_ Elsterales* (1.79%), *norank_f_ Gemmatimonadaceae* (1.51%), *norank_f_norank_ o_norank_c_TK10* (1.38%), *Bryobucter* (1.26%), *Actinospica* (1.20%), *norank_f_ norank_ o_ norank_ c_ AD3* (1.10%), *Jatrophihabitans* (1.10%), and *Mycobacterium* (1.09%) were the dominant soil bacterial genera of the bulk soil.

All these results indicated that the soil bacterial community composition in the rhizosphere varies significantly between red amaranth and green amaranth. Among them, *Streptomyces, Pseudonocardia*, *Pseudolabrys*, *Acidibacter, norank_ f_ Micropepsaceae, Bradyrhizobium*, and *Nocardioides* were the unique dominant soil bacterial genera in the rhizosphere of red amaranth. In contrast, *Conexibacter*, *norank_f_norank_o_norank_c_TK10* and *norank_f_ norank_o_ norank_ c_AD3* were the unique dominant soil bacterial genera in the rhizosphere of green amaranth.

In addition, as shown in Figure 3c, the numbers of dominant soil fungal phyla (those with an abundance greater than 1%) in the rhizosphere of red and green amaranths and bulk soil were 4, 4, and 2, respectively. First, the dominant soil fungal phyla in the rhizosphere of red amaranth were Ascomycota (66.68%), Basidiomycota (16.35%), unclassified _ k _ Fungi (9.94%), and Chytridiomycota (1.84%). In contrast, the main dominant soil fungal phyla in the green amaranth treatment were Ascomycota (71.85%), Basidiomycota (14.64%), unclassified_k_Fungi (15.55%), and Chytridiomycota (1.03%). The main dominant soil fungal phyla in the bulk soil treatment were Ascomycota (78.63%) and Basidiomycota (20.08%).

At the genus level, the numbers of dominant soil fungal genera (with an abundance greater than 1%) in the rhizosphere of red and green amaranths and bulk soil were 13, 13, and 15, respectively (Figure 3d).

First, *unclassified_ k_ Fungi* (15.55%), *Saitozyma* (12.90%), *unclassified_f_Microascaceae* (10.79%), *Microuscus* (7.39%), *Fusarium* (6. 66%), *Trichoderma* (5.77%), *Talaromyces* (5.19%), *Penicillium* (2.24%), *Cadophialophora* (3.09%), *Neocosmospora* (2.48%), *Chaetomium* (2.46%), *Coniosporium* (2.44%), *unclassified_c_Sordariomycetes* (2.22%), and *Aspergillues* (1.02%) were the dominant soil fungal genera in the rhizosphere of the red amaranth.

In contrast, *Saitozyma* (15.36%), *Microuscus* (13.17%), *Chaetonium* (12.06%), *unclassified_f_Microascaceae* (9.73%), *unclassified_K Fungi* (9.94%), *Talaromyces* (6.96%), *Fusarium* (3.92%), *Penicillium* (3.35%), *Aspergillues* (2.96%), *Trichoderma* (2.86%), *Cladophialophora* (1.85%), *Coniosporium* (1.40%), *Urificified_ c_ Sordariomycetes* (1.17%), and *Neocosmospora* (1.05%) were the dominant soil fungal genera in the rhizosphere of green amaranth.

Additionally, *Penicillium* (22.40%), *Saitozyma* (18.26%), *unclassified_f_Microascaceae* (7.43%), *Clonostachys* (6.04%), Talaromyees (5.74%), *Talaromyces* (5.74%), *Fusarium* (5.01%), *Trichoderma* (4.73%), *Cludophialophora* (2.98%), *Neocosmospora* (2.70%), *Coniosporium* (2.31%), *unclassified_c Sordariomycetes* (2.05%), *Chordomyces* (1.87%), *Chaetomium* (1.29%), and *Chaetosplaeria* (1.03%) were the dominant soil fungal genera in the bulk soil.

All of the above results suggested that the composition of the dominant soil fungi at the genus level did not change in the rhizosphere of red or green amaranths. However, the abundances of the dominant soil fungal genera in the rhizosphere differed between red and green amaranths. In comparison with those of green amaranth, the abundances of *unclassified_f_Microascaceae*, *Fusarium*, *Trichoderma*, *Cladophialophora*, *Neocosmospora*, *Coniosporium*, and *unclassified_c_Sordariomycetes* were greater, and the abundances of *Saitozyma*, *Penicillium*, *Microascus*, *Aspergillues, Talaromyces*, and *Chaetomium* were lower in the rhizosphere of red amaranth.

### 3.4. LEfSe Analysis of Soil Bacterial Communities in the Rhizospheres of Red and Green Amaranth Plants

**Figure 4:**
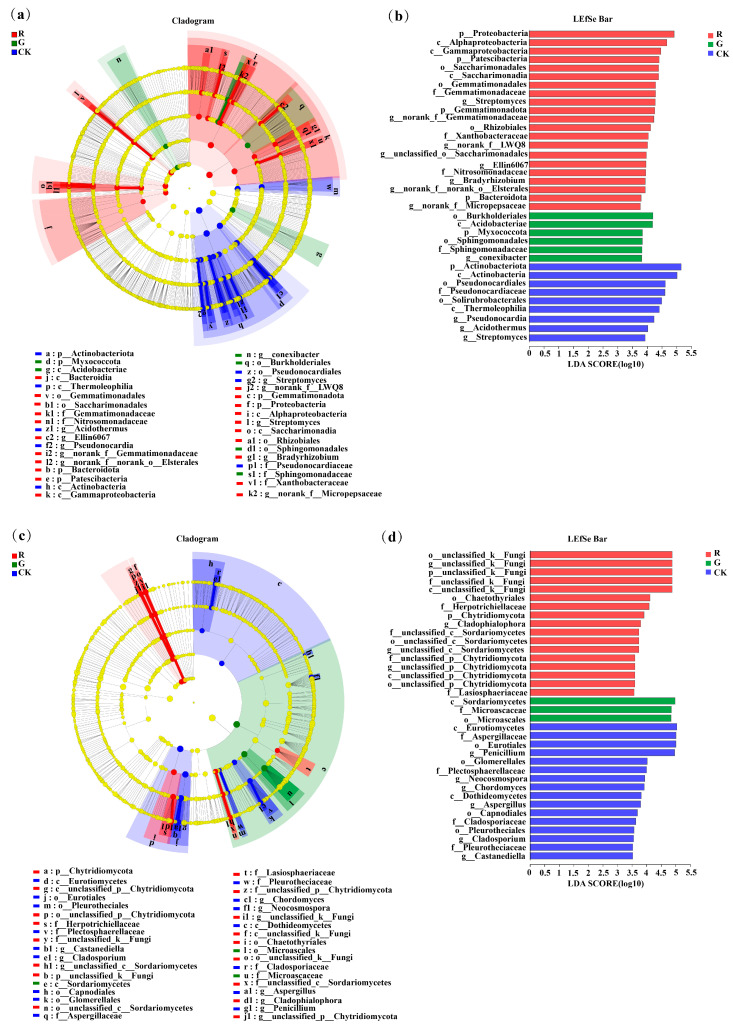
LEfSe analysis of soil bacterial (**a**) and fungi (**b**) communities in the rhizospheres of red and green amaranth plants.Score plots of bacterial (**c**) and fungi (**d**) communities in the rhizospheres of red and green amaranth plants.Pathologically, nodes indicate microbial taxa that are significantly enriched in the corresponding group and have a significant effect on the differences between groups (p, phylum; C, class; 0, order; f, family; and g, genus). (*p* < 0.05, LDA scores ≥ 3.5).

As shown in Figure 4a, LEfSe analysis at the phylum-to-genus level was also conducted to identify bacteria with significant differences in abundance in the rhizospheres of red and green amaranths and in the bulk soil. The results showed that 35 bacterial branches were significantly different (LDA > 3.5, *p* < 0.05). In particular, at the phylum level, Proteobacteria, Patescibacteria, Gemmatimonadota, and Bacteroidota were significantly enriched as the dominant soil bacterial phyla in the rhizosphere of red amaranth. In contrast, Myxococcota was significantly enriched as the dominant soil bacterial phylum in the rhizosphere of green amaranth. In addition, at the genus level, *Streptomyces, norank_f__Gemmatimonadaceae*, *Bradyrhizobium, norank_f__LWQ8*, *unclassified_o__Saccharimonadales*, *Ellin6067*, *norank_f__Micropepsaceae* and *norank_f __norank_o__ElsteralesH* were significantly enriched as the dominant soil bacterial genera in the rhizosphere of red amaranth; in contrast, *Conexibacter was* significantly enriched as the dominant soil bacterial genus in the rhizosphere of green amaranth.

Moreover, 35 fungal branches were also significantly different according to LEfSe analysis (Figure 4c,d). At the phylum level, unclassified_k__Fungi and Chytridiomycota were significantly enriched as the dominant soil fungal phyla in the rhizosphere of red amaranth. Additionally, at the genus level, *unclassified_k__Fungi*, *Fusarium*, *Cladophialophora*, *unclassified_c__Sordariomycetes* and *unclassified_p__Chytridiomycota* were significantly enriched as the dominant soil fungal genera in the rhizosphere of red amaranth. In contrast, *Aspergillues* was significantly enriched as the dominant soil fungal genera in the rhizosphere of green amaranth.

### 3.5. Analysis of the Collinearity of Soil Bacterial and Fungi Communities in the Rhizosphere of Red and Green Amaranth Plants

**Figure 5:**
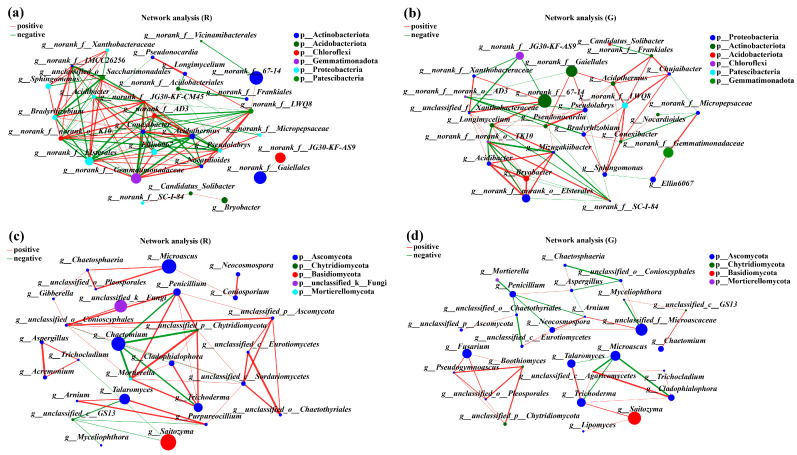
Collinear network analysis of the soil bacterial (**a**,**b**) and fungi (**c**,**d**) communities in the rhizospheres of red and green amaranths. Different levels are indicated by different prefixes (p, phylum; g, genus). The size of the nodes in the graph indicates the size of the species abundance, and different colors indicate different species; the colors of the connecting lines indicate positive and negative correlations, with red indicating positive correlation and green indicating negative correlation (*p* < 0.05); the thickness of the lines indicates the size of the correlation coefficient; the coarser the line is, the greater the correlation between the species; and the greater the number of lines is, the closer the connection between the species and other species.

To describe the potential relationships of soil microbial communities in the rhizospheres of red and green amaranths, network analysis was constructed based on Spearman correlation coefficients. The topological characteristics of the soil bacteria in the rhizospheres of red and green amaranths were shown in Table 2. In comparison with green amaranth, the Average Degree (avgk) and Average Path Distance (GD) in rhizospheres of red amaranth were all increased. However, the Average Clustering Coefficient (avgCC) and Modularity were decreased. Meanwhile, a total of 108 edges were detected in rhizospheres of red amaranth; 53 edges were positively correlated and 55 edges were negatively correlated. Among them, *norank_f__norank_o__norank_c__TK10*, *norank_f__Gemmatimonadaceae*, *norank_f__norank_o__Elsterales*, *Acidothermus*, and *Conexibacter* showed the strongest correlations between each other. In green amaranth, a total of 90 edges were detected, and 45 edges showed positive correlations and 25 edges revealed negative correlations. Meanwhile, *Longimycelium*, *norank_f__SC-I-84*, *Mizugakiibacter*, *norank_f__norank_o__Elsterales*, and *Acidibacter* were the five bacteria most strongly correlated with other bacteria in rhizospheres of green amaranth (Figure 5a,b).

For soil fungi, in comparison with green amaranth, the Average Degree (avgk), Average Clustering Coefficient (avgCC), and Average Path Distance (GD) were all increased in rhizospheres of red amaranth. Also, a total of 52 edges were detected in rhizospheres of red amaranth, of which 40 were positively correlated, and 12 were negatively correlated. The five fungal groups most strongly associated with other soil fungi in rhizospheres of red amaranth were *unclassified_c__Sordariomycetes*, *unclassified_o__Conioscyphales*, *Penicillium*, *Mortierella*, and *unclassified_p__Chytridiomycota*. By contrast, 41 edges were detected in rhizospheres of green amaranth. Among them, 28 and 13 edges showed positive and negative correlations, respectively. Moreover, the five fungal groups most strongly associated with other soil fungi in rhizospheres of green amaranth were *Trichoderma*, *Penicillium*, *Arnium*, *unclassified_f__Microascaceae*, and *unclassified_c__Eurotiomycetes* (Figure 5c,d).

### 3.6. Functional Analysis of Soil Bacterial and Fungi Communities in the Rhizospheres of Red and Green Amaranth Plants

**Figure 6:**
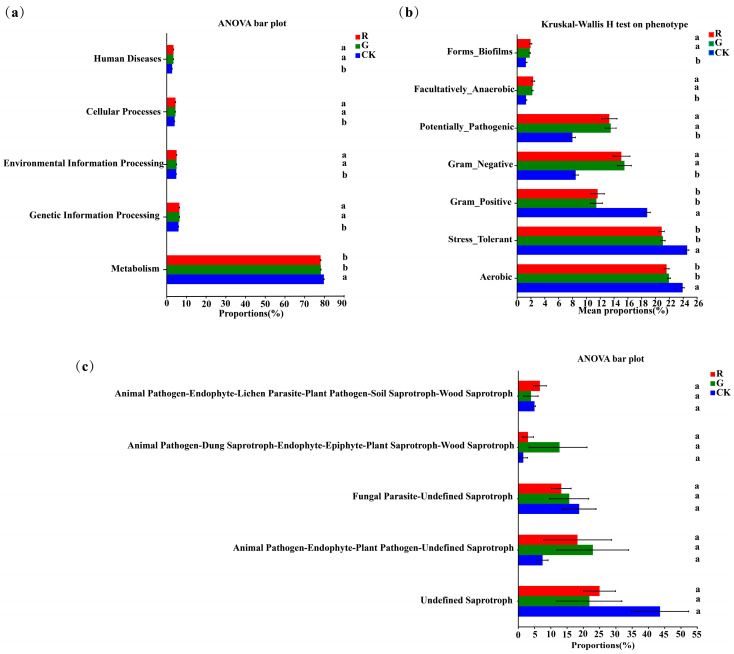
Functional analysis of soil bacteria (**a**,**b**) and fungi (**c**) in the rhizospheres of red and green amaranths. Different lowercase letters indicate significant differences between soil microbes of different amaranth color varieties (*p* < 0.05).

PICRUSt analysis was also carried out to predict bacterial functions in the rhizospheres of red and green amaranths. At the operational taxonomic unit (OTU) level, no significant changes were detected in functional genes between red and green amaranths (Figure 6a). Furthermore, significant differences of the bacterial community phenotypes were also not found between red and green amaranths. However, the Aerobic, gram_positive and stress_tolerant functions of soil bacteria were significantly higher in bulk soil than those in the rhizospheric soil of red and green amaranths. Additionally, gram_negative, forms_biofilms, facultatively_anaerobic, and potentially_pathogenic were significantly higher in the rhizospheric soils of red and green amaranths than those of bulk soil (Figure 6b).

FUNGuild (Fungi Functional Guild) fungal communities can be classified by a microecological guild (Figure 6c). The Undefined Saprotroph, Animal Pathogen-Endophyte—Plant Pathogen-Undefined Saprotroph, Fungal Parasite-Undefined Saprotroph, Animal Pathogen-Dung Saprotroph-Endophyte-Epiphyte-Plant Saprotroph-Wood Saprotroph, and Animal Pathogen-Endophyte-Lichen Parasite-Plant Pathogen-Soil Saprotroph-Wood Saprotroph were the dominant fungal functional groups in the rhizosphere of red and green amaranths and bulk soil. However, for soil fungal functions, there was no significant difference in the rhizosphere between red and green amaranths.

## 4. Discussion

Plant traits are tightly coupled with soil bacterial composition [44]. Previous research on how plant sex influences the microbiome assembly in dioecious plants has found that male and female plants have different root exudates, which recruit different rhizosphere bacteria. This may ultimately reflect differences in their root morphology and physiological characteristics [45,46]. For example, male plants typically secreted higher levels of specific compounds, such as certain phenolics or growth hormones, which could enrich specific bacterial communities [47]. In contrast, female plants tended to secrete higher levels of organic acids or amino acids, which might be more suitable for the growth and reproduction of certain growth-promoting bacteria [48,49]. Additionally, soil microorganisms affected the plant traits through the production of phytohormones, which could enhance plant antioxidant capacity, and could improve plant uptake of certain types of micronutrients [50,51,52]. For instance, phosphorus solubilizing bacteria (e.g., Bacillus megateriumand Pseudomonas fluorescens) could improve phosphorus uptake by plants, which could indirectly affect carotenoid synthesis and change the leaves’ color [53]. Meanwhile, rhizobacteria (e.g., Trichoderma and Pseudomonas) could promote chlorophyll synthesis and improve the leaves’ green color by increasing nitrogen levels in legumes [54]. Moreover, microorganisms could enhance the antioxidant capacity of plants and reduce the damage of leaf pigmentation under stresses (e.g., drought, salinity, high temperature) [55]. In our study, although significant differences of soil microbial diversity and richness in rhizospheres could not be detected, the soil bacterial and fungal compositions significant altered in the rhizospheres of red and green amaranths. This indicates that different color amaranths exactly recruited different soil bacteria and fungi in rhizospheres.

A higher degree and lower modularity of the soil microbial symbiotic network tended to be a whole function interacting with microorganisms complexly [56,57]. Meanwhile, the complexity of the network is often positively correlated with community stability [58]. Our network analysis also indicated that a higher degree and clustering coefficient could be found in the rhizospheres of red amaranth compared to those of green amaranth. It also indicated that higher stability and more functions of soil microbial community rhizospheres of red amaranth could be detected compared to those of green amaranth.

At the phylum level, LEfSe analysis found that Proteobacteria, Patescibacteria, Gemmatimonadota, and Bacteroidota were significantly enriched in red amaranth rhizosphere soil (*p* < 0.05); Myxococcota was significantly enriched in green amaranth soil. Proteobacteria are oligotrophic denitrifying bacteria that utilize a variety of carbon sources, promote plant growth, and are found in a variety of ecosystems and biological microcosms [59]. Chen et al. [60] have shown a significant negative correlation between Proteobacteria and abscisic acid. In addition, Ascomycota and Basidiomycota were the dominant fungi phyla in the rhizospheres of red and green amaranth, and the proportion of Basidiomycota in the rhizosphere of red amaranth increased. Interestingly, Timoneda et al. [24] found that Basidiomycota can produce betalains.

At the genus level, *Streptomyces*, *Pseudonocardia*, *Pseudolabrys*, *Acidibacter*, *norank_ f_ Micropepsaceae*, and *Bradyrhizobium* were the unique dominant soil bacterial genera in the rhizosphere of red amaranth. In contrast, *Conexibacter*, *norank_f_norank_o_norank_c_TK10* and *norank_f_ norank_o_ norank_ c_AD3* were the unique dominant soil bacterial genera in the rhizosphere of green amaranth. The physiological and molecular genetic analysis of ethylene biodegradation by Nocardia species conducted by previous researchers has confirmed that Nocardia can produce ethylene [61]. Cytokinins could also promote the accumulation of betatin pigments in plants under either dark or light conditions [62]. *Bradyrhizobium* can not only produce indium-3-acetic acid (IAA) but also induce an increase in the intracellular Ca^2+^ concentration in plant cells [63,64]. It is reported that auxin (IAA) can increase the level of a plant’s free tyrosine and strongly promote the accumulation of betatin [65]. Wang et al. [66] reported that Ca^2+^ can be directly or indirectly involved in betalain biosynthesis. In addition, in comparison with green amaranth, we also found a significant increase in the proportion of streptomyces in red amaranth (P < 0.05). *Streptomyces* gene sequencing identified a large number of cytochrome P450 genes, which are involved in the biosynthesis of secondary metabolites [20]. Previous research on the catalytic products of cytochrome P450 enzymes in Streptomyces has found that cytochrome P450 genes are key genes in the betaine metabolic pathway and play an important role in the biosynthesis of betalains [67,68].

Meanwhile, *unclassified_k__Fungi*, *Fusarium*, *Cladophialophora*, *unclassified_c__Sordariomycetes*, and *unclassified_p__Chytridiomycota* were significantly enriched as the dominant soil fungal genera in the rhizosphere of red amaranth. In contrast, *Aspergillues* was significantly enriched as the dominant soil fungal genera in the rhizosphere of green amaranth. Warhade et al. [69] reported a positive effect of *Fusarium* on the biosynthesis of betalain pigments. Abdul et al. [70] found that gibberellins can be produced by *Aspergillues* and *Penicillium*. However, gibberellic acid (GA3) inhibits the synthesis of betalain [71]. In this experiment, we found that *Penicillium* decreased in the rhizosphere of red amaranth compared to green amaranth. In addition, the abundance of *Trichoderma* in the rhizosphere soil of red amaranth was about twice as high as that of green amaranth. Segarra et al. [72] reported that plant defense-protective hormones, such as jasmonic acid, are widely present in *Trichoderma*. Previous research on the synthesis of betalains in Portulaca oleracea stimulated by exogenous methyl jasmonate and other inducers has shown that jasmonic acid and methyl jasmonate can effectively promote the accumulation of betalains in the plant [73]. These results suggest that *Fusarium* and *Trichoderma* are enriched in the rhizosphere of red amaranth and are most likely involved in the synthesis and accumulation of pigments in red amaranth. In contrast, *Aspergillues* and *Penicillium* were enriched in the rhizosphere soil of green amaranth, probably inhibiting betatin synthesis.

All of the above results suggested that different color amaranths exactly recruited different soil microbes. Among them, soil microorganisms, in association with betain synthesis, were significantly enriched in the rhizospheres of red amaranth. By contrast, soil microorganisms, in relation to betain synthesis inhibition, were also significantly enriched in the rhizospheres of green amaranth.

## 5. Conclusions

Soil microorganisms in the rhizosphere of red amaranth and green amaranth were significant. Among them, soil microorganisms promoting betatin synthesis were enriched in the rhizosphere of red amaranth. For example, Nocardioides, Bradyrhizobium, Streptomyces, Fusarium, and Trichoderma microorganisms closely related to betatin synthesis or accumulation were enriched in the rhizosphere of red amaranth, which may promote color formation. On the contrary, soil microorganisms that inhibit betatin synthesis are enriched in the rhizosphere of green amaranth, such as Aspergillues and Penicillium inhibiting the synthesis of betatain enrichment in the rhizosphere of green amaranth. The above results suggest that different genotypes of amaranth species, especially red amaranth, need to recruit some unique rhizosphere bacteria and fungi to promote their own color formation. However, the mechanism of association between microorganisms and the host is complex. The function of microorganisms involved in amaranth color formation is still unknown, and further studies are needed to elucidate this mechanism of action.

## Figures and Tables

**Table 1:** Soil bacterial and fungi diversity and richness in the rhizosphere of red and green amaranths.

Classifications	Treatments	Shannon	Simpson	Ace	Chao1	Coverage
Bacteria	R	5.94 ± 0.30 a	0.0090 ± 0.0025 a	3120.82 ± 394.09 a	3110.46 ± 430.23 a	0.98
	G	6.13 ± 0.24 a	0.0073 ± 0.0024 a	3368.23 ± 191.08 a	3376.82 ± 187.12 a	0.98
	CK	5.77 ± 0.07 a	0.013 ± 0.001 a	3031.67 ± 143.63 a	3022.41 ± 324.16 a	0.98
Fungi	R	5.94 ± 0.30 a	0.0090 ± 0.0025 a	3120.82 ± 394.09 a	3110.46 ± 430.23 a	0.99
	G	6.13 ± 0.24 a	0.0073 ± 0.0024 a	3368.23 ± 191.08 a	3376.82 ± 187.12 a	0.99
	CK	5.77 ± 0.07 a	0.012 ± 0.001 a	3031.67 ± 143.63 a	3022.41 ± 324.16 a	0.99

Note: The data in the table are presented as the means ± SDs. Different lowercase letters indicate significant differences between soil microbes of different amaranth color varieties (*p* < 0.05).

**Table 2:** Topological characteristics of the red and green amaranth network analysis.

Classifications	Treatments	Average Degree (avgk)	Average Clustering Coefficient (avgCC)	Average Path Distance (GD)	Modularity
Soil bacteria	R	7.45	0.54	10.83	0.71
	G	5.19	0.57	9.05	2.15
Soil fungi	R	3.78	0.56	7.39	0.58
	G	3.04	0.47	4.59	3.13

## Data Availability

The original reads were stored in the NCBI Sequence Read Archive (SRA) database (accession number: SUB14314150).
